# Integrated genomics approach to identify biologically relevant alterations in fewer samples

**DOI:** 10.1186/s12864-015-2138-4

**Published:** 2015-11-14

**Authors:** Pratik Chandrani, Pawan Upadhyay, Prajish Iyer, Mayur Tanna, Madhur Shetty, Gorantala Venkata Raghuram, Ninad Oak, Ankita Singh, Rohan Chaubal, Manoj Ramteke, Sudeep Gupta, Amit Dutt

**Affiliations:** Advanced Centre for Treatment, Research and Education in Cancer, Tata Memorial Center, Navi Mumbai, Maharashtra 410210 India; Department of Medical Oncology, Tata Memorial Hospital, Tata Memorial Center, Mumbai, Maharashtra India

## Abstract

**Background:**

Several statistical tools have been developed to identify genes mutated at rates significantly higher than background, indicative of positive selection, involving large sample cohort studies. However, studies involving smaller sample sizes are inherently restrictive due to their limited statistical power to identify low frequency genetic variations.

**Results:**

We performed an integrated characterization of copy number, mutation and expression analyses of four head and neck cancer cell lines - NT8e, OT9, AW13516 and AW8507-- by applying a filtering strategy to prioritize for genes affected by two or more alterations within or across the cell lines. Besides identifying *TP53*, *PTEN*, *HRAS* and *MET* as major altered HNSCC hallmark genes, this analysis uncovered 34 novel candidate genes altered. Of these, we find a heterozygous truncating mutation in Nuclear receptor binding protein, *NRBP1* pseudokinase gene, identical to as reported in other cancers, is oncogenic when ectopically expressed in NIH-3 T3 cells. Knockdown of *NRBP1* in an oral carcinoma cell line bearing *NRBP1* mutation inhibit transformation and survival of the cells.

**Conclusions:**

In overall, we present the first comprehensive genomic characterization of four head and neck cancer cell lines established from Indian patients. We also demonstrate the ability of integrated analysis to uncover biologically important genetic variation in studies involving fewer or rare clinical specimens.

**Electronic supplementary material:**

The online version of this article (doi:10.1186/s12864-015-2138-4) contains supplementary material, which is available to authorized users.

## Background

Head and neck squamous cell carcinoma (HNSCC) is the sixth-most-common cancer worldwide, with about 600,000 new cases every year, and includes cancer of the nose cavity, sinuses, lips, tongue, mouth, salivary glands, upper aerodigestive tract and voice box [1]. Recent large scale cancer genome sequencing projects have identified spectrum of driver genomic alterations in HNSCC including *CDKN2A*, *TP53*, *PIK3CA*, *NOTCH1*, *HRAS*, *FBXW7*, *PTEN*, *NFE2L2*, *FAT1*, and *CASP8* [2–4]. These landmark studies apply elegant statistical methodologies like MutSig [5], Genome MuSiC [6], Intogen [7], InVEx [8], ActiveDrive [9] and GISTIC [10] in identifying significantly altered genes across large sample cohorts by comparing rate of mutations of each gene with background mutation rate to determine an unbiased enrichment-- a minimum ~150 patients or higher is required for identification of somatic mutations of 10 % population frequency in HNSCC [11]. These genome-wide analysis may not be directly applicable for studies involving fewer or rare clinical specimen that are inherently restrictive due to the limited statistical power to detect alterations existing at lower frequency.

On the other hand, given that a cancer gene could be selectively inactivated or activated by multiple alterations, an integrative study design performed by combining multiple data types can potentially be helpful to achieve the threshold for statistical significance for studies involving fewer or rare clinical specimen. For example, a tumor suppressor gene-- deleted in 1 % of patients, mutated in another 3 %, promoter-hypermethylated in another 2 % and out of frame fused with some other chromosomal region in 2 %-- may be considered to be altered with a cumulative effect of 8 % based on integrative analysis [12, 13]. Combinatorial sources of genetic evidence converging at same gene or signalling pathway can also limit false positives by filtering strategy and potentially reducing the multiple hypothesis testing burden for identification of causal genotype-phenotype associations [14]. Using similar approaches for posterior refinement to indicate positive selection, Pickering et al. identified four key pathways in oral cancer by integrating methylation to copy number variation and expression [15]; and, more recently, Wilkerson et al. proposed superior prioritisation of mutations based on integrated analysis of the genome and transcriptome sequencing than filtering based on conventional quality filters [16]. These and several other reports all together emphasize integration of multi-platform genomic data for identification of cancer related genes [17].

Here, we perform characterization of four head and neck cancer cell lines, established from Indian head and neck cancer patients, using classical cytogenetic approach, SNP arrays, whole exome and whole transcriptome sequencing. Next, we apply the widely used posterior filtering strategy of results obtained from genome wide studies to effectively reduce the amount of data obtained from individual platforms. Adopting such an integrative approach allow us to identify biological relevant alterations affected by two or more events even from fewer samples.

## Methods

### Cell culturing and single cell dilution for establishing clonal cells

Four HNSCC tumor cell lines established within Tata Memorial Center from Indian patients and described before were acquired: NT8e, OT9, AW13516, AW8507 [18, 19]. All the cell lines were maintained in DMEM media (Gibco, USA). For clonal selection, growing culture was trypsinized and diluted as 1 cell per 100 ml of media and dispensed in a 96 well plate with follow up subculture of clones that survived.

### SNP array analysis

Genomic DNA was extracted from pre-clonal and clonal cell lines using PAXgene Tissue DNA Kit (Qiagen, USA). 200 ng of good quality DNA from each sample was submitted to Sandor Proteomics (Hyderabad, India) for sample preparation and genome wide SNP array using Illumina Infinium assay (Human660W-quad BeadArray chip) following manufacturer’s standard protocol. Array data was pre-processed using GenomeStudio (Illumina Inc., USA) for quality control check. To retain only good quality genotyping calls, a threshold GenCall score of 0.25 was used across all samples. A total of 396, 266 SNPs were retained after this filtering. These SNPs were then used for copy number analysis using Genome Studio plug-in cnvPartition 3.2 and an R package Genome Alteration Print (GAP) [20]. Inferred copy numbers were then annotated with genomic features using BedTools (v. 2.17.0) [21]. Copy number segments of more than 10 Mb in size were classified as arm-level amplifications and were identified as non-significant alterations. Focal amplifications (less than 10 Mb) were used for further analysis.

### Cytogenetic karyotyping

Cells grown in complete media (60–70 % confluent) were treated with colcemid (0.1 ng/ul, Sigma, USA) to arrest them in metaphase. After incubation of 6 h at 37oC and trypsinisation, cells were washed with pre-warmed KCl (0.075 M) (Sigma, USA) and incubated with KCl at 37 °C in water bath for 60 min. After the incubation is over, cells were fixed with Carnoy’s fixative solution on pre chilled microscopic glass slides (chilled in alcohol) by pipette around 70 μl of cell suspension, drop by drop from height (50 cm). Slides were kept on the water bath at 70 °C for few seconds followed by drying on heating block (set at 80 °C). Metaphase of cells was confirmed by observing chromosomes using a phase contrast inverted microscope (Zeiss, USA). Confirmed metaphase captured cells were aged by keeping the slides at 60 °C for 3 h followed by trypsin digest (Trypsin/EDTA - concentration of 0.025 %, Sigma, USA). Giemsa stain (Sigma, USA) (3 %) was applied using coplin Jar for 15 min on slides followed by washing with distilled water.

### Exome sequencing

Exome enrichment was performed using manufacturer’s protocol for Illumina TruSeq exome enrichment kit in which 500 ng of DNA libraries from six samples were pooled to make total 3 μg DNA mass from which 62 MB of targeted exonic region covering 20,976 genes was captured. Exome enriched library was quantified and validated by real-time PCR using Kappa quantification kit at the Next-Generation Genomics Facility (NGGF) at Center for Cellular and Molecul ar Platforms (CCAMP, India). Whole exome libraries of AW13516, AW8507 and OT9 were loaded onto Illumina HiSeq 1000 for 2 X 100 bp paired-end sequencing with expected coverage of ~ 100 X. NT8e cell line was sequenced with 2 X 54 bp paired-end and 2 X 100 bp paired-end sequencing. Raw sequence reads generated were mapped to NCBI human reference genome (build GRCh37) using BWA v. 0.6.2 [22]. Mapped reads were then used to identify and remove PCR duplicates using Picard tools v. 1.74 (http:broadinstitute.github.io/picard/). Base quality score recalibration and indel re-alignment were performed and variants were called from each cell line separately using GATK v. 1.6-9 [23, 24] and MuTect v. 1.0.27783 [25]. All the variants were merged and dumped into local MySQL database for advanced analysis and filtering. We used hard filter for removing variants having below 5X coverage to reduce false positives. For cell lines we use dbSNP (v. 134) [26] as standard known germline variants database and COSMIC (v. 62) [27] as standard known somatic variants database. Variants identified in cell lines, which are also there in dbSNP but not in COSMIC were subtracted from the database. Remaining variants were annotated using Oncotator (v. 1.0.0.0rc7) [28], and three functional prediction tools PolyPhen2 (build r394) [29], Provean (v. 1.1) [30] and MutationAccessor (release 2) [31]. Variants found deleterious by any two out of three tools were prioritized. Variants having recurrent prediction of deleterious function were prioritized. Variants from exome sequencing were compared to variants identified from transcriptome sequencing for cross-validation using in-house computer program.

### Transcriptome sequencing

Transcriptome libraries for sequencing were constructed according to the manufacturer’s protocol. Briefly, mRNA was purified from 4 μg of intact total RNA using oligodT beads (TruSeq RNA Sample Preparation Kit, Illumina). 7 pmol of each library was loaded on Illumina flow cell (version 3) for cluster generation on cBot cluster generation system (Illumina) and clustered flow cell was transferred to Illumina HiSeq1500 for paired end sequencing using Illumina paired end reagents TruSeq SBS Kit v3 (Illumina) for 200 cycle. De-multiplexing was done using CASAVA (version 1.8.4, Illumina). Actively expressed transcripts were identified from sequencing data by aligning them to the reference genome hg19 using Tophat (v. 2.0.8b) [32] and quantifying number of reads per known gene using cufflinks (v.2.1.1) pipeline [33]. All the transcripts were then binned by log_10_(FPKM + 1) to differentiate the significantly expressed transcripts from the background noise. Since paired normal of these cell lines cannot be obtained, we defined significant change in expression for those genes whose expression is higher (>60 %) or lower (<40 %) than the median expression as suggested in [34]. Gene set enrichment was performed by submitting actively expressed transcripts lists to MSigDB V4 [35] and filtering resulting gene lists by *p*-value of enrichment. Variants were identified from transcriptome sequencing using GATK [23, 24]. Only variants having overlap with exome sequencing were considered as true genomic variants. Fusion transcripts were identified using ChimeraScan (v.0.4.5) [36]. Candidate fusion events supported by minimum 10 read pairs were used for integration and visualization in Circos plot.

### Integrated analysis

Genes identified to be altered by SNP array, transcriptome sequencing and exome sequencing were then used for integrative analysis to prioritize the genes which are harbouring multiple types of alteration in same or different cell line. Gene level converging of genomic data were emphasized in identification of biologically relevant alterations across platform and samples. Taking this into consideration, we designed gene prioritization based on three steps: 1) selection of genes harbouring positive correlation of focal copy number and gene expression; 2) selection of genes harbouring point mutations with detectable transcript and or altered copy number, and 3) selection of genes harbouring multiple type of alterations identified from above two gene lists (Additional file 1: Figure S7). Circos plot representation of integrated genomics data was generated using Circos tool (v. 0.66) [37].

### Sanger sequencing validation

PCR products were purified using NucleoSpin Gel and PCR Clean-up kit (MACHEREY-NAGEL) as per manufacture’s protocol and quantified using Nano-Drop 2000c Spectrophotometer (Thermo Fisher Scientific Inc.) and submitted for sequencing in capillary electrophoresis 3500 Genetic Analyzer (Life Technologies). Sanger sequencing traces were analysed for mutation using Mutation Surveyor [38]. The details of all the primers used for mutation analysis have been provided in Additional file 2: Table S7.

### DNA copy number validation

Quantitative-real time PCR and data analysis was performed using Type-it® CNV SYBR® Green PCR (cat. No. 206674) as per manufacturer’s instructions on 7900HT Fast Real-Time PCR System. The details of all the primers used for DNA copy number analysis have been provided in Additional file 2: Table S8.

### RNA extraction, cDNA synthesis, quantitative real time PCR

Total RNA was extracted from cell lines using RNeasy RNA isolation kit (Qiagen) and Trizol reagent (Invitrogen) based methods and later resolved on 1.2 % Agarose gel to confirm the RNA integrity. RNA samples were DNase treated followed (Ambion) by first strand cDNA synthesis using Superscript III kit (Invitrogen) and semi-quantitative evaluative PCR for GAPDH was performed to check the cDNA integrity. cDNA was diluted 1:10 and reaction was performed in 10 μl volume in triplicate. The melt curve analysis was performed to check the primer dimer or non-specific amplifications. Real-time PCR was carried out using KAPA master mix (KAPA SYBR® FAST Universal q PCR kit) as per manufacturer’s instructions in triplicate on 7900HT Fast Real-Time PCR System. All the experiments were repeated at least twice independently. The data was normalized with internal reference *GAPDH*, and analysed by using delta-delta Ct method described previously [39]. The details of all the primers used for expression analysis have been provided in Additional file 2: Table S8.

### Generation of p BABE-*NRBP1*-PURO constructs

The cDNA of Human *NRBP1* was amplified from AW13516 cell line using Superscript III (Invitrogen, cat no 18080–093) in a TA cloning vector pTZ57R/T(InsTAclone PCR cloning kit, K1214, ThermoScientific), later site-directed mutagenesis was done using QuikChange II Site-Directed Mutagenesis Kit (cat.no. 200523) as per manufacturer’s instructions. Later both wild type and mutant *NRBP1* cDNA sequenced confirmed using Sanger sequencing and were sub-cloned in to retroviral vector *p* BABE-puro using restriction digestion based cloning (SalI and BamHI).

### Generation of stable clone of NIH-3 T3 overexpressing *NRBP1*

Two hundred ninety-three T cells were seeded in 6 well plates one day before transfection and each constructs (pBABE-puro) along with pCL-ECO helper vector were transfected using Lipofectamine LTX reagent (Invitrogen) as per manufacturer’s protocol. Viral soup was collected 48 and 72 h post transfection, passed through 0.45 μM filter and stored at 4 °C. Respective cells for transduction were seeded one day before infection in six well plate and allowed to grow to reach 50–60 % confluency. One ml of the virus soup (1:5 dilution) and 8ug/ml of polybrene (Sigma) was added to cells and incubated for six hours. Cells were maintained under puromycin (Sigma) selection.

### shRNA mediated knockdown of *NRBP1* in HNSCC cells

We retrieved shRNA sequences targeting human NRBP1 from TRC (The RNAi Consortium) library database located in sh1 (3′ UTR) and sh2 (CDS). Target sequences of NRBP1 shRNA constructs: sh1 (TRCN0000001437), 5′-CCCTCTGCACTTTGTTTACTTCT-3′; sh2 (TRCN0000001439), 5′-TGTCGAGAAGAGCAGAAGAATCT-3′. shGFP target sequences is 5′-GCAAGCTGACCCTGAAGTTCAT-3′. p-LKO.1 GFP shRNA was a gift from David Sabatini (Addgene plasmid # 30323) [40]. Cloning of shRNA oligos were done using AgeI and EcoRI restriction site in p-LKO.1 puro constructs. Bacterial colonies obtained screened using PCR and positive clone were sequence verified using Sanger sequencing. Lentiviral production and stable cell line generation performed as described earlier [41]. In brief, Lentivirus were produced by transfection of shRNA constructs and two helper vector in 293 T cells as described [42]. Transduction was performed in HNSCC cells by incubating for 6 h in presence of 10 μg/ml polybrene and post infection media was replaced with fresh media. Puromycin selection was performed two days post infection in presence of 1 μg/ml. Puromycin selected cells were harvested and total cell lysate prepared and expression of NRBP1 was analysed using anti-NRBP1 antibody (Santa Cruz Biotechnology; sc-390087) and GAPDH (Santa Cruz Biotechnology; sc-32233).

### Soft Agar colony formation assay

The cells were harvested 48 h after transfection, and an equal number of viable cells were plated onto soft agar after respective treatments for determination of anchorage-independent growth. For analysis of growth in soft agar, 5 × 10^3^cells were seeded in triplicate onto a six well dish (Falcon) in 3 ml of complete medium containing 0.33 % agar solution at 37 °C. Cells were fed with 500 μl of medium every 2 days. From each well randomly 10 field images were taken using Phase contrast Inverted microscope (Zeiss axiovert 200 m) and colonies were counted manually.

Growth curve analysis - 25,000 cells/well were seeded in 24 well plates and growth was assessed post day 2, 4 and 6 by counting the cells using a haemocytometer. Percent survival were plotted at day 4 relative to day 2 and later normalized against scrambled or empty vector control.

### Western blot analysis

Cells were lysed in RIPA buffer and protein concentration was estimated using BCA method [43]. 50 and 100 μg protein was used for NIH-3 T3 and HNSCC cell lines western analysis. The protein was separated on 10 % SDS-PAGE gel, transfer was verified using Ponceau S (Sigma), transferred on nitrocellulose membrane and blocked in Tris-buffered saline containing 5 % BSA (Sigma) and 0.05 % Tween-20(Sigma). Later, blots were probed with anti-NRBP1 (Santa Cruz Biotechnology; sc-390087), anti-total ERK1/2 (Cell signaling; 4372S), anti-Phospho ERK1/2 (Cell signaling; 4370S) and anti- GAPDH antibody (Santa Cruz Biotechnology; SC-32233). The membranes were then incubated with corresponding secondary HRP-conjugated antibodies (Santa Cruz Biotechnology, USA) and the immune complexes were visualized by Pierce ECL (Thermo Scientific, USA) according to manufacturer’s protocol. Western blot experiments were performed as independent replicates.

### Statistical analysis

Chi-square and t-test were performed using R programming language and GraphPad Prism. A *p*-value cut-off of 0.05 was used for gene expression, copy number and variant analysis.

### Availability of supporting data

All genomics data have been deposited at the ArrayExpress (http://www.ebi.ac.uk/arrayexpress/), hosted by the European Bioinformatics Institute (EBI), under following accession numbers: E-MTAB-3958 : Whole transcriptome data; E-MTAB-3961 : Whole exome data; and, E-MTAB-3960 : SNP array data

## Results

We characterized genetic alterations underlying four head and neck cancer cell lines followed by TCGA dataset to identify cumulative significance of biologically relevant alterations by integrating copy number, expression and point mutation data.

### Characterization of four HNSCC cell lines established from Indian patients

Given that higher accumulative effect of individual genes can be reckoned by integrative analysis, we argue that these alteration can possibly be determined even with fewer samples. As a proof of principle, we performed an integrated characterization of karyotype analysis, copy number analysis, whole transcriptome and exome sequencing of 4 HNSCC cell lines established from Indian patients. In brief, significantly altered chromosomal segments based on copy number analysis were filtered based on nucleotide variant information and aberrant expression of transcripts to allow prioritization of regions harboring either deleterious mutation or expressing the transcript at significantly high levels, in addition to the stringent intrinsic statistical mining performed for each sample.

#### Karyotype analysis

**Fig. 1:**
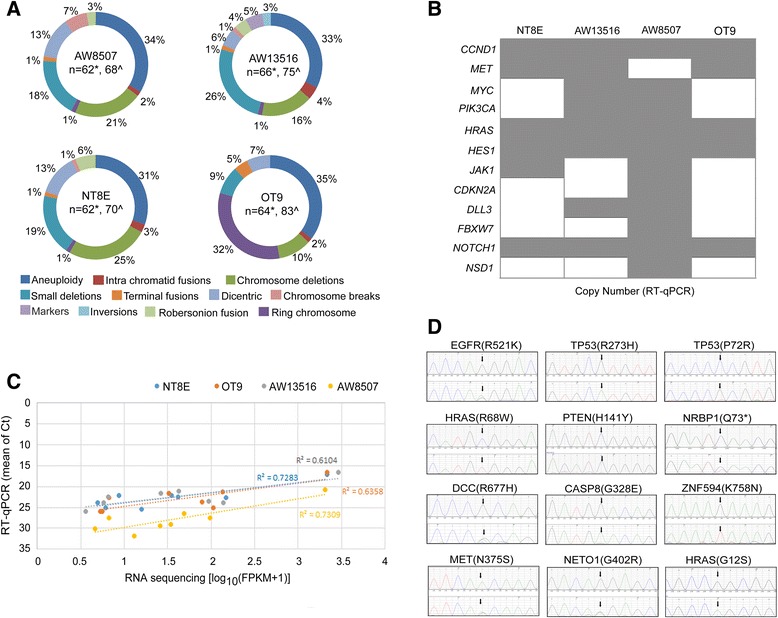
Genomic alterations identified in HNSCC cell lines. **a** Chromosomal aberrations identified by 25 independent karyotype of each cell line is represented in circular form. Chromosome numbers in each cell line are indicated by n, as observed from karyotype (*) and predicted by SNP array (^). **b** Heatmap representation of copy number changes in key hallmark genes identified by SNP array and validated by qPCR. Grey box indicates validated and white box indicates invalidated copy number change in respective cell line. **c** Scatter plot representation of hallmark gene’s expression. X-axis shows gene expression quantified by RNA sequencing and Y-axis shows gene expression quantified by RT-qPCR. Genes showed positive correlation between RNA sequencing and RT-qPCR. **d** Several high-quality point mutations identified by exome sequencing were validated by Sanger sequencing. Sanger sequencing trace were visualized using Mutation Surveyor software showing reference sequence trace in upper panel and mutant sequencing trace in below panel. Arrows represent mutation position with reference and mutated base indicated above the arrow

The hyperploidy status of AW13516, AW8507, NT8E and OT9 cell lines were inferred by classical karyotyping with an average ploidy of 62, 62, 66 and 64, respectively that were largely consistent with ploidy as inferred form SNP array analysis (Fig. 1a; Additional file 1: Figure S1) and as reported for tumor cells lines [18, 19]. We specifically observed dicentric and ring chromosomes at elevated frequency indicating higher chromosomal instability (CIN) [44]. Overall distribution of chromosomal aberrations in each HNSCC cell lines showed similar pattern, representing an overall similar genomic structure of all HNSCC cell lines.

#### Copy number analysis

We performed genotyping microarray using Illumina 660 W quad SNP array chips of all the cell lines (Additional file 1: Figure S2). After stringent filtering of initial genotyping calls, on an average, 253 genomic segments of copy number changes were obtained per cell line. By limiting segment size at 10 Mb an average 166 focal segments were identified, including loss of copy number and LOH at 3p which is known to have correlation to advanced stage of tumor progression and poor clinical outcome [45, 46]; copy number gain on 11q known to be associated with advanced stage, recurrence and poor clinical outcome [47]; LOH at 8p and 9p which are known to be associated with advanced stage and survival [48] (Additional file 2: Table S1); and amplification of known oncogenes *EGFR* in AW13516, OT9; *MYC* in AW13516 and AW8507 cells; *JAK1* in NT8E, AW8507; *NSD1* in AW8507; and *MET* in AW13516 and OT9 (Additional file 2: Table S2). Several hallmark genes were found to be amplified in cell lines such as *CCND1, NOTCH1,* and *HES1* in all four cells; *PIK3CA* in AW13516, AW8507; deletion of *CDKN2A* in AW13516; *FBXW7* in NT8E, AW13516 and OT9 cells were detected and validated by real time PCR (Fig. 1b, Additional file 2: Table S2) in each cell line.

#### Whole transcriptome analysis

Whole transcriptome sequencing revealed 17,067, 19,374, 16,866 and 17,022 genes expressed in AW13516, AW8507, NT8e and OT9 respectively. Total ~5000 transcripts having less than 0.1 log_10_(FPKM + 1) were filtered out because of biologically non-significant expression level (Additional file 1: Figure S3). The upper quartile (>60 %) was considered as highly expressed genes and lower quartile (<40 %) was considered as lowly expressed genes. Gene set enrichment analysis of upper quartile showed enrichment of genes (data not shown) known to be up regulated in nasopharyngeal carcinoma [49]. All the transcripts showed 75 % overlap of expression profile with each other (Additional file 1: Figure S4) indicating overall similar nature of cell lines. Over expression of hallmark of HNSCC such as *CCND1, MYC, MET, CTNNB1, JAK1, HRAS, JAG1*, *and HES1* and down regulation of *FBXW7, SMAD4* in at least 3 cell line were observed and validated by quantitative real time PCR (Additional file 2: Table S3). A positive correlation was observed between transcriptome FPKM and qPCR Ct values (Fig. 1c).

#### Analysis of mutational landscape

All the cell lines were sequenced for whole exome at about 80X coverage using Illumina HiSeq. The relative coverage of each coding region was comparable across all four cell lines (Additional file 2: Table S4; Additional file 1: Figure S5). The coding part of the four cell line genome consist 28813, 47892, 20864 and 25029 variants in AW13516, AW8507, NT8e and OT9 cell line, respectively. Filtering of known germline variants (SNPs) and low quality variants left 5623, 4498, 2775, 5139 non-synonymous variants in AW13516, AW8507, NT8e and OT9 cell line, respectively (Additional file 2: Table S4). Of 20 HNSCC hallmark variants predicted as deleterious by two of three algorithms used for functional prediction [29–31], 17 variants could be validated by Sanger sequencing (Fig. 1d; Additional file 2: Table S5) including: *TP53* (R273H), *TP53* (P72R), *PTEN* (H141Y), *EGFR* (R521K), *HRAS* (G12S and R78W), and *CASP8* (G328E).

### Integrated analysis identifies hallmark alterations in HNSCC cell lines

**Fig. 2:**
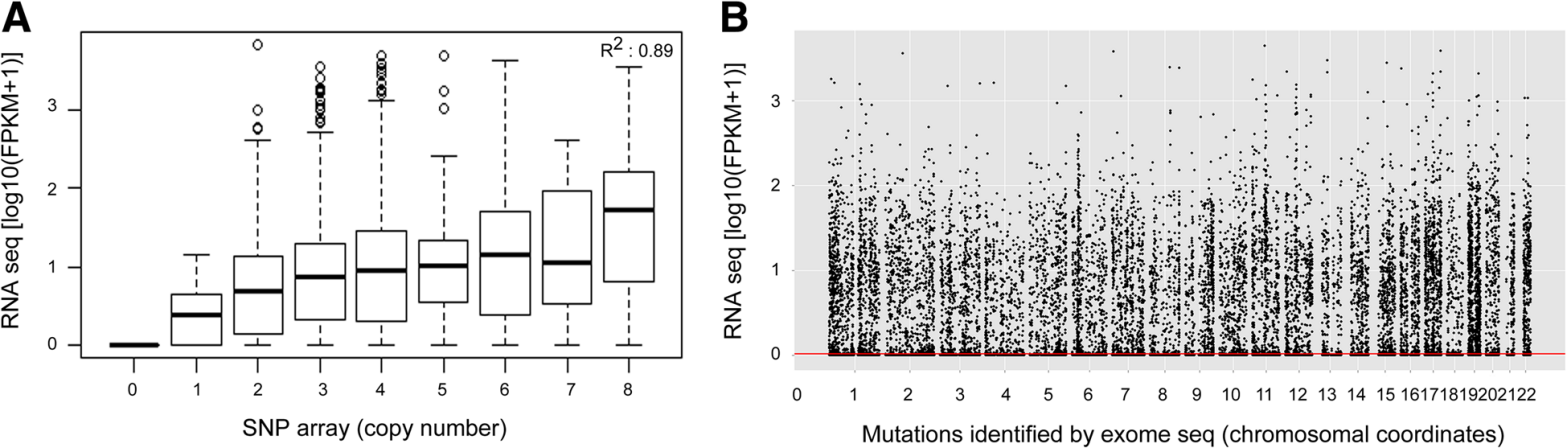
Integration of copy-number, gene expression and single nucleotide variants. **a** Box plot representations showing focal copy number variations identified by SNP array on X-axis and gene expression quantified by RNA sequencing on Y-axis. Focally amplified or deleted genes from all four cell lines showed a positive trend with over expression or under expression respectively. **b** Scatter plot representation of genes harboring single nucleotide variants and it’s expression from all four cell lines. Variants identified by whole exome sequencing are displayed by genomic coordinates on X-axis and normalized quantity of gene expression (by transcriptome sequencing) is displayed on Y-axis. Red line denotes 0.01 log10(FPKM + 1) filter which removes ~50 % of the total variants

**Fig. 3:**
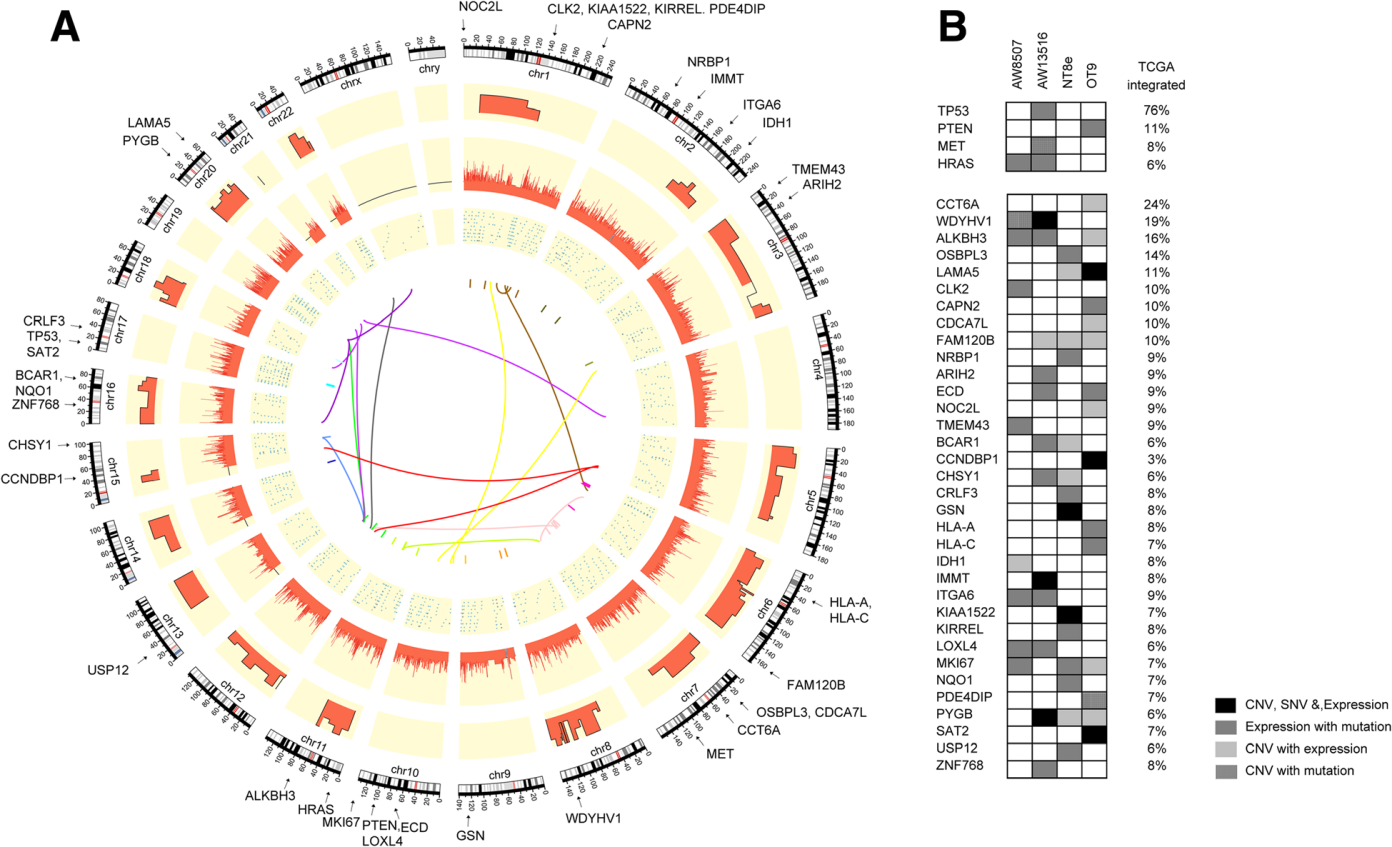
Integrative genomic landscape of HNSCC. **a** Circos plot representations of genes identified by integrated genomic analysis of HNSCC cell lines. From outside to inside: karyotype, CNVs, Gene expression (FPKM), SNVs and translocations. Red colour indicates copy number gain or higher gene expression and blue colour indicates copy number loss or lower gene expression in CNV and FPKM tracks respectively. Non-synonymous mutations are indicated as blue triangles and grey circles represents non-sense mutations in SNV track. Fusion transcripts identified by transcriptome sequencing are shown as arc coloured by their chromosome of origin identified by ChimeraScan. **b** Heatmap representation of novel genes identified by integrated analysis of four HNSCC cell lines and their incidence in 279 HNSCC samples from TCGA study. Amplification (red) and deletions (blue) are indicated by filled box, over expression (red) and under expression (blue) are indicated by border line to the box, mis-sense (green), non-sense (black) and in-frame (brown) mutations are indicated by smaller square box

The first step of integration analysis involved identification of genes with positively correlated copy number and expression data. While no significant correlation was observed among expression and arm-level copy number segments (Additional file 1: Figure S6a), median expression of focally amplified and deleted genes positively correlated to their expression (Fig. 2a and Additional file 1: Figure S6b). About 1000 genes with focal copy number changes with consistent expression pattern were identified from four cell lines. The second step of integration analysis involved identification of mutated genes that were expressed. Number of missense mutations identified from transcriptome sequencing (67,641 variants) were much higher than from exome sequencing (30,649 variants). Filtering of exome variants against transcriptome variants reduced total number of 9253 unique missesne variants in all four cell lines (Fig. 2b). Two thousand four hundred seventy-nine missense mutations of 9523 total mutations found across all cells were used for further integration with copy number and expression data (Additional file 1: Figure S7). Next, as third step of integration, we sorted genes with altered copy number, expression levels and harboring non-synonymous mutations for integrated analysis based on criterion as described in methodology in four cell lines (Additional file 1: Figure S7). Briefly, genes harbouring two or more type of alterations were selected: harbouring positive correlation of focal copy number and gene expression; or those harbouring point mutations with detectable transcript harbouring the variant—based on which, we identified 38 genes having multiple types of alterations (Additional file 2: Table S6). These include genes known to have somatic incidences in HNSCC: *TP53*, *HRAS*, *MET* and *PTEN*. We also identified *CASP8* in AW13516 cell line which was recently identified as very significantly altered by ICGC-India team in ~50 Indian HNSCC patients [50]. We additionally identified novel genes like *CCNDBP1, GSN, IMMT, LAMA5, SAT2* and *WDYHV1* to be altered by all three analysis i.e. CNV, expression and mutation. These all genes were also found to be altered in TCGA dataset with minimum 3 % cumulative frequency (Additional file 1: Figure S8). The overall convergence of copy number, expression and mutation data in each cell line is represented as circos plot (Fig. 3a; Additional file 1: Figure S9). Among the novel genes identified, of genes with at least one identical mutation previously reported include a pseudokinase *Nuclear receptor binding protein NRBP1* harboring heterozygous truncating mutation (Q73*) in NT8e cells, identical to as reported in lung cancer and altered in other cancers [51, 52].

### Mutant *NRBP1* is required for tumor cell survival and is oncogenic in NIH3T3 cells

**Fig. 4:**
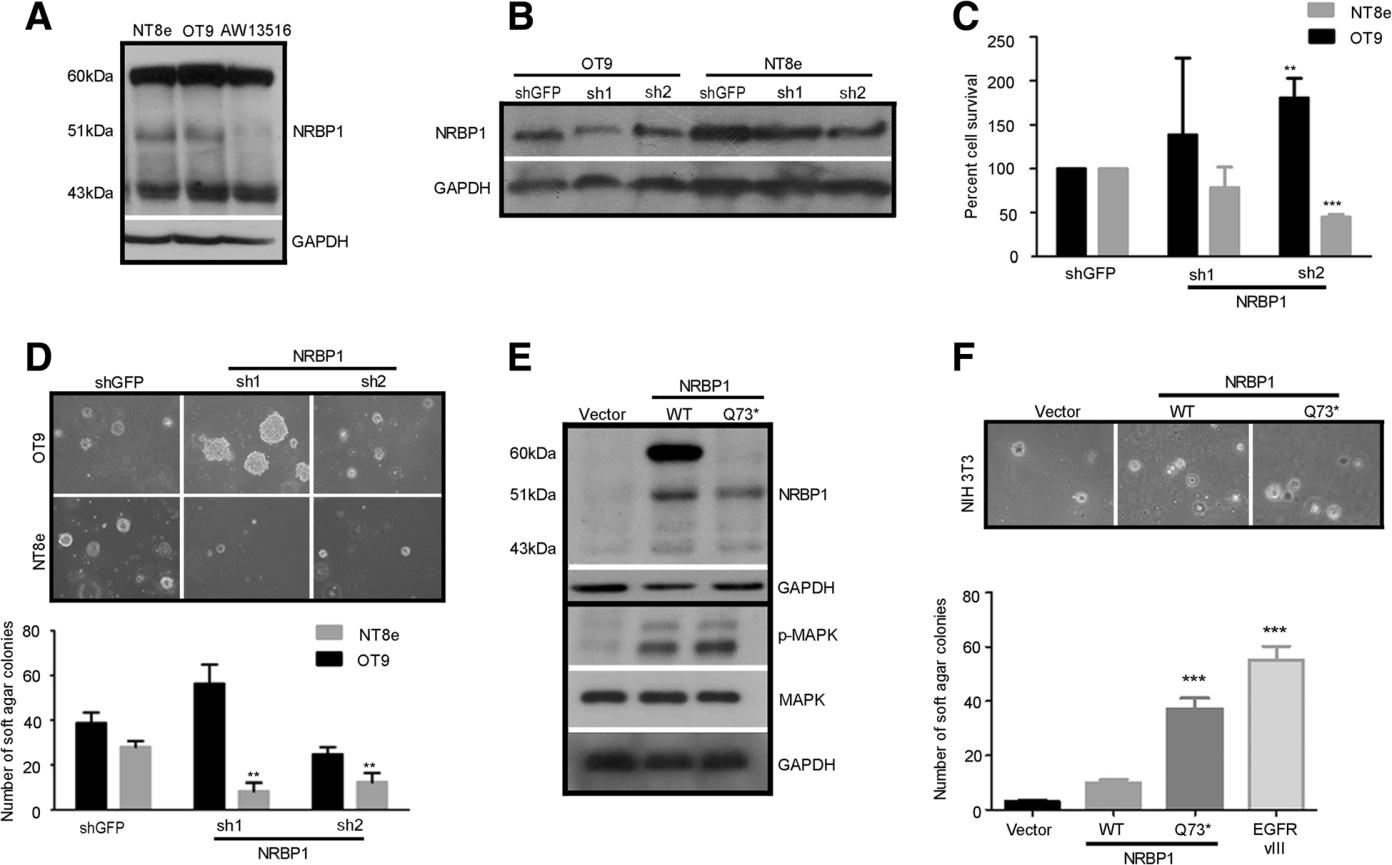
Mutant *NRBP1* is required for tumor cell survival and is oncogenic in NIH3T3 cells. Knockdown of mutant *NRBP1* expression with shRNA inhibits transformation and survival of HNSCC cell lines. **a** Western blot analysis of total NRBP1 expression level in HNSCC cell lines. *NRBP1* encodes for three different nuclear receptor binding protein isoform: 60 kDa, 51 kDa and 43 kDa in NT8e cells (lane 1), OT9 cells (lane 2). AW13516 cells express only two isoforms (lane 3). **b** Western blot representation of *NRBP1* partial knockdown by two independent hairpins (shNRBP1#1 and shNRBP1#2) in NT8e and OT9 cells. The hairpin constructs inhibit cell survival as assessed by cell counting as described in methods. **c** and anchorage-independent growth as assessed by colony formation in soft agar **d** in the NT8e cells harboring an NRBP1 Q73* mutation, but not the OT9 cells, which express WT *NRBP1*. shGFP, a hairpin specific for green fluorescent protein, was used as a negative control. All results are normalized to survival or colony formation by cells infected with empty vector. Images were taken at 10X magnification. **e** Western blot analysis of *NRBP1* wild type and mutant (Q73*) in NIH-3 T3 cells. Vector control, NIH-3 T3 clones overexpressing wild-type and mutant *NRBP1* is shown. *NRBP1* Q73* mutation expresses 51 kDa isoform, while two isoform of 60 kDa and 51 kDa can be seen in 3 T3 clones overexpressing wild-type *NRBP1*. Western blot analysis of total and phosphorylated MAPK in NIH-3 T3 clones expressing *NRBP1* wild type (lane 2) and mutant NRBP1 (Q73*). **f** Representative images (taken at 20x) of soft agar colony formed by NIH-3 T3 cells expressing *NRBP1* wild type and Q73*. Bar graph representation of number of soft agar colonies formed by stable NIH-3 T3 clones. NIH-3 T3 cells expressing *EGFR* VIII was used as positive control and relative comparison of transforming ability of mutant *NRBP1*. *** *P*-value <0.0001, ** *P*-value <0.001

*NRBP1* encodes for three different nuclear receptor binding protein isoform using three alternative translational initiation sites of 60 kDa, 51 kDa and 43 kDa [53], as were observed in 2 of 3 HNSCC cells (Fig. 4a). To determine whether expression of mutant *NRBP1* is required for tumor cell survival, we tested shRNA constructs in two HNSCC cells expressing all three forms of WT *NRBP1* (OT9 cells) and mutant *NRBP1* (NT8e cells). We demonstrate that even partial knockdown of mutant *NRBP1* expression in the NT8e cells, but not WT *NRBP1* expression in the OT9, significantly inhibited anchorage-independent growth and cell survival (Fig. 4b–d). We next tested the oncogenic role of *NRBP1*. mRNAs harboring premature termination (nonsense) codons are selectively degraded by Nonsense-mediated mRNA decay (NMD) [54]. However, mRNAs with nonsense mutations in the first exon are known to bypass NMD [55]. When ectopically expressed in NIH-3 T3 cells, mutant *NRBP1* transcript escape non-sense mediated degradation as determined by real time PCR (Additional file 1: Figure S10). All three isoform of NRBP1 were detected in NIH-3 T3 cells expressing wild type *NRBP1* cDNA. However, only two isoform of 51 kDa and 43 kDa were detected in cells transfected with mutant *NRBP1* cDNA (Fig. 4e upper panel). The over expression of the mutant *NRBP1* in NIH3T3 cells conferred anchorage-independent growth, forming significantly higher colonies in soft agar than cells expressing wild type *NRBP1* (Fig. 4f). Transformation of NIH-3 T3 cells by *NRBP1* over expression was accompanied by elevated phosphorylation of the MAPK (Fig. 4e lower panel).

### Integrated analysis of TCGA dataset for HNSCC hallmark genes

**Fig. 5:**
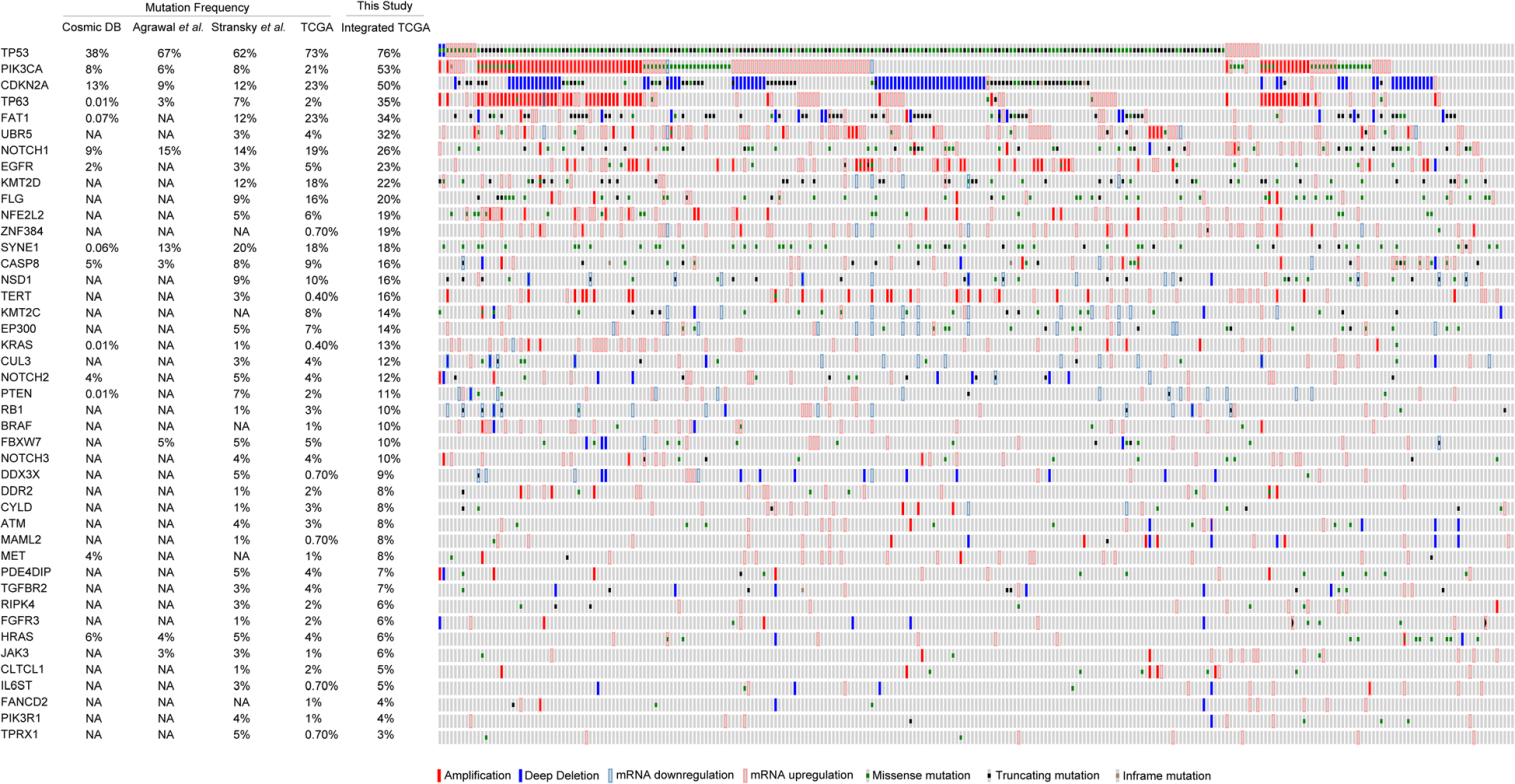
Integrative genomic landscape of HNSCC tumors in TCGA dataset. Heatmap representation of hallmark genes in 279 HNSCC samples from TCGA study with frequency of alterations based on integrated CNVs, gene expression and SNVs. Frequency of alterations in the gene based on Cosmic database, Agrawal et al*.*, Stransky et al*.* and TCGA study are also shown for comparison with integrated analysis. Amplification (red) and deletions (blue) are indicated by filled box, over expression (red) and under expression (blue) are indicated by border line to the box, mis-sense (green), non-sense (black) and in-frame (brown) mutations are indicated by smaller square box

Next, as a proof of principle, we computated cumulative frequency of copy number variations, expression changes and point mutations across 43 genes with ~3 % and higher mutation frequency in HNSCC TCGA dataset. As expected and described for few genes [4, 56], most of the genes were found to be altered at higher cumulative incidence than as reckoned by individual alterations (Fig. 5). Interestingly, three class of hallmark genes involved in HNSCC could be distinctly identified: genes that are primarily altered by mutations like *TP53* and *SYNE1*; genes that are sparsely altered by amplification or overexpression in addition to mutations like *FAT1*, *NOTCH1*, *KMT2D*, and *FLG*; and, genes that are preferentially altered by amplification or over expression over point mutations with higher cumulative effect than known before. Of these, previously described genes like *PIK3CA*, *CDKN2A*, *TP63*, *EGFR*, *CASP8*, *NFE2L2*, and *KRAS* show more than twice cumulative effect of alteration while rest of the genes are altered at several folds higher cumulative frequency based on integrated analysis. Furthermore, three genes-- *UBR5*, *ZNF384* and *TERT* were found to be altered with cumulative frequency of 32, 19, and 16 %, respectively that has not been previously described in HNSCC.

## Discussion

We have characterized genetic alterations of unknown somatic status underlying four head and neck cancer cell lines of Indian origin patient by subjecting them to a thorough karyotype based characterization, SNP array based analysis, whole exome capture sequencing, and mRNA sequencing.

Integrated analysis of the cell lines establish their resemblance to primary tumors. Consistent with literature, most frequent copy number gains in head and neck cancer cells in this study were observed at 2q, 3q, 5p and 7p, and deletions at 3p, 9p, 10p, 11q, 14q, 17q and 19p, as reported earlier [57, 58]. Integration of multiple platform with the copy number variation, allowed us to identify the functionally relevant alterations including several hall marks genes known to be involved in HNSCC, viz. *PIK3CA, EGFR, HRAS, MYC, CDKN2A, MET, TRAF2, PTK2* and *CASP8*. Of the novel genes, *JAK1* was found to be amplified in two of the cell lines and overexpressed in all 4 HNSCC cells; *NOTCH1* known to harbor inactivating mutations in HNSCC [3, 50] was found to be amplified in all 4 and overexpressed in 2 of 4 HNSCC cells, known to be play dual role in a context dependent manner [59].

We also observed missense mutations in several novel genes such as *CLK2, NRBP1, CCNDBP1, IDH1, LAMA5, BCAR1, ZNF678,* and *CLK2*. Of these, genes with at least one identical mutation previously reported include *NRBP1* (Q73*), a pseudo kinase, found in NT8e cells, earlier reported in lung and other cancers [51, 52], with an overall 9 % cumulative frequency alteration in TCGA HNSCC dataset (Additional file 1: Figure S8). Of 48 pseudo kinases known in human genome, several have been shown to retain their biochemical catalytic activities despite lack of one or more of the three catalytic residues essential for its kinase activity, with their established roles in cancer [60–62].

Interestingly, several activating mutant alleles of *NRBP1 homolog Drosophila Madm* (Mlf1 adapter molecule) *3 T4* (Q46*); *2U3* (C500*); *3G5* (Q530*); *7 L2* and *3Y2* (that disrupts splice donor site of first exon) are known, wherein alternative translation start codons is similarly suggestive for a varying degree of pinhead phenotype severity associated with the mutant alleles [53, 63]. Studies in the fruit fly have provided important insights into mechanisms underlying the biology of growth promoting *NRBP1 homolog Drosophila Madm*. A recent study suggests *Drosophila Madm* interacts with *Drosophila bunA* that encodes a gene homologous to human *Transforming Growth Factor-β1 stimulated clone-22 TSC-22* [63]; that were later shown to interact even in mammalian system [64]. Interestingly, mammalian tumor suppressor *TSC-22* is known to play an important role in maintaining differentiated phenotype in salivary gland tumors [65], a subtype of head and neck cancer. More recently, studies have shown poor clinical outcomes are associated with *NRBP1* over expression in prostate cancer [64]. We provide the first functional analysis of mutant *NRBP1* and establish that NIH-3 T3 cells expressing the mutant *NRBP1* enhance their survival and anchorage independent growth, while its knock down diminishes survival and anchorage-independent growth by oral cancer cells expressing activating *NRBP1* mutations. Thus, NT8e cells harboring mutant *NRBP1* was found to be consistent with its suggestive role in prostate cancer biology and other model organisms. Interestingly, *NRBP1* has also been shown to be involved in intestinal progenitor cell homeostasis with tumor suppressive function [66], suggesting its role is specific to the cellular context. This study identifies *NRBP1* mutant to play an oncogenic role in head and neck cancer. However, in depth systematic sequencing of *NRBP1* in a wide variety of tumor types may help indicate utility of NRBP1 inhibition in human cancer.

Furthermore, based on TCGA data integrated analysis, cumulative alteration frequency of *TP63* (35 %), *EGFR* (23 %) and *NFEL2* (19 %) were found to be higher than reported in COSMIC and cBioPortal, consistent with as described in other reports [4, 56]. Of alterations not defined before, *UBR5*, *ZNF384* and *TERT* were found to be altered at higher frequency at 32, 19, 16 %, respectively. Interestingly, recurrent *UBR5-ZNF384* fusion has been shown to be oncogenic in EBV-associated nasopharyngeal subtype of HNSCC [67]; amplification of *TERT* has been shown to be higher in lung squamous [68], suggesting these alterations as potential squamous specific event, though that warrants detailed systematic assessment.

In overall, this study underscores integrative approaches through a filtering strategy to help reckon higher cumulative frequency for individual genes affected by two or more alterations to achieve the threshold for statistical significance even from fewer samples. The integrative analysis as described here, in essence, is based on a linear simplified assumption of disease aetiology that variation at DNA level lead to changes in gene expression causal to transformation of the cell. As a major deficiency, only genes that are subject to multiple levels of biological regulation are likely to be determined by this approach than genes that are primarily altered by single alteration like amplification or over expression.

## Conclusion

As a proof of principle, integrated analysis of copy number variation, exome and transcriptome of 4 head and neck cancer cell lines and TCGA HNSCC dataset identify *NRBP1, UBR5*, *ZNF384* and *TERT* as novel candidate oncogenes in HNSCC. However, systematic functional experimental validation is required to further guide and identify true driver events of these alterations. Additionally, the genetically- defined cellular systems characterized by integrated genomics analysis in this study (NT8e, OT9, AW13516, AW8507), together with the identification of novel actionable molecular targets, may help further facilitate the pre-clinical evaluation of emerging therapeutic agents in head and neck cancer.

## Additional files

Additional file 1:
**Additional Figures S1-S10.** Chromosomal aberration in HNSCC patient derived cell lines AW8507, AW13516, NT8e and OT9. (A) Representative karyotype of AW8507, AW13516, NT8e and OT9 cells is shown from total 25 karyotypes obtained per cell line. (B) Chromosomal aberrations identified by 25 independent karyotype of each cell line is represented in circular form. Chromosome numbers in each cell line are indicated by n, as observed from karyotype (*) and predicted by SNP array (^). Copy number changes in HNSCC cell lines identified by SNP array. Genome alteration print (GAP) of (A) AW8507, (B) AW13516, (C) NT8e AND (D) OT9 cell lines obtained by SNP array. First horizontal block represents B-allele frequency, second block represents absolute copy number, third block is log R ratio. Frequency of transcripts per binned log transformed FPKM + 1. Raw RNA sequencing data was binned to obtain frequency of genes per log10(FPKM + 1) in (A) AW8507, (B) AW13516, (C) NT8e and (D) OT9. Horizontal dotted lines indicates percentile of transcripts in the quadrant. Similarity of gene expression in HNSCC cell lines. Number of genes commonly expressed between AW8507, AW13516, NT8e and OT9 cell lines. Relative depth in exome sequencing. Relative depth of sequencing for various genomic regions in (A) AW8507, (B) AW13516, (C) NT8e and (D) OT9. Correlation of copy number with gene expression. (A) Arm level and (B) focal copy number changes and gene expression (y-axis) are shown for AW8507, AW13516, NT8e and OT9 cell lines. Correlation of focal copy number with gene expression was 1.5 fold higher in AW8507, 5.2 fold higher in AW13516, 2.4 fold higher in NT8e and 1.6 fold higher in OT9 cell line compare to arm level copy number changes. *P*-value cut-off of 0.05 was used as threshold for statistical significance.* denotes *P*-value <0.05, ** <0.005, *** <0.0005. Schematic view of data reduction in integrated genomic analysis. Flow chart depicts the reduction of data at each stage of integration. First row indicates number of genes identified from each platform as raw calls. Second and third row indicates number of genes left after each step of integration with main selection parameter indicated outside the box. Integrative genomic alterations of genes in TCGA dataset of HNSCC tumors. Heatmap representation of 38 genes in 279 HNSCC samples from TCGA study with frequency of alterations based on integrated CNVs, gene expression and SNVs. Amplification (red) and deletions (blue) are indicated by filled box, over expression (red) and under expression (blue) are indicated by border line to the box, mis-sense (green), non-sense (black) and in-frame (brown) mutations are indicated by smaller square box. Circos plot representation of HNSCC cell lines. Circos plot representations of integrated genomic data of (A) AW8507, (B) AW13516, (C) NT8e and (D) OT9 cell lines. From outside to inside: karyotype, CNVs, Gene expression (FPKM), SNVs and translocations. Red colour indicates copy number gain or higher gene expression and blue colour indicates copy number loss or lower gene expression in CNV and FPKM tracks, respectively. Non-synonymous mutations are indicated as blue triangles and grey circles represents non-sense mutations in SNV track. Fusion transcripts identified by transcriptome sequencing are shown as arc coloured by their chromosome of origin identified by ChimeraScan. *NRBP1* expression in NIH-3 T3 cells. qPCR analysis of *NRBP1* gene expression in NIH-3 T3 stably expressing wild and mutant. Data was normalized against *GAPDH* and fold change plotted. *P* value <0.0001 is denoted as ***. (PDF 9017 kb) Additional file 2:
**Additional Tables S1-S8:** Copy number alterations of known genomic locations identified in HNSCC cell lines. Copy number alterations in halmark genes identified in HNSCC cell lines. Gene expression of hallmark genes by RNA sequencing and qPCR. Features of whole exome and transcriptome sequencing. Validation of mutations in hallmark and novel genes. Details of mutations identified by integrated analysis in HNSCC cell lines. Primer sequences used for Sanger sequenicng based validation of mutations. Primers used for copy number and gene expression study using qPCR. (ZIP 249 kb)

## Abbreviations

HNSCC: Head and neck squamous cell carcinoma
COSMIC: Catalogue of Somatic Mutation In Cancer
TCGA: The Cancer Genome Atlas
ICGC: International Cancer Genome Consortium
CIN: Chrmosomal Instability
CNV: Copy Number Variation
