# Supplementary material for: Integrated genomics approach to identify biologically relevant alterations in fewer samples
Source: BMC Genomics. 2015 Nov 14;16:936. doi: 10.1186/s12864-015-2138-4 (PMC4647579; doi:10.1186/s12864-015-2138-4)
Supplement: Additional file 1: — Additional Figures S1-S10. Chromosomal aberration in HNSCC patient derived cell lines AW8507, AW13516, NT8e and OT9. (A) Representative karyotype of AW8507, AW13516, NT8e and OT9 cells is shown from total 25 karyotypes obtained per cell line. (B) Chromosomal aberrations identified by 25 independent karyotype of each cell line is represented in circular form. Chromosome numbers in each cell line are indicated by n, as observed from karyotype (*) and predicted by SNP array (^). Copy number changes in HNSCC cell lines identified by SNP array. Genome alteration print (GAP) of (A) AW8507, (B) AW13516, (C) NT8e AND (D) OT9 cell lines obtained by SNP array. First horizontal block represents B-allele frequency, second block represents absolute copy number, third block is log R ratio. Frequency of transcripts per binned log transformed FPKM + 1. Raw RNA sequencing data was binned to obtain frequency of genes per log10(FPKM + 1) in (A) AW8507, (B) AW13516, (C) NT8e and (D) OT9. Horizontal dotted lines indicates percentile of transcripts in the quadrant. Similarity of gene expression in HNSCC cell lines. Number of genes commonly expressed between AW8507, AW13516, NT8e and OT9 cell lines. Relative depth in exome sequencing. Relative depth of sequencing for various genomic regions in (A) AW8507, (B) AW13516, (C) NT8e and (D) OT9. Correlation of copy number with gene expression. (A) Arm level and (B) focal copy number changes and gene expression (y-axis) are shown for AW8507, AW13516, NT8e and OT9 cell lines. Correlation of focal copy number with gene expression was 1.5 fold higher in AW8507, 5.2 fold higher in AW13516, 2.4 fold higher in NT8e and 1.6 fold higher in OT9 cell line compare to arm level copy number changes. P-value cut-off of 0.05 was used as threshold for statistical significance.* denotes P-value <0.05, ** <0.005, *** <0.0005. Schematic view of data reduction in integrated genomic analysis. Flow chart depicts the reduction of data at each stage of integration. Fir [file 12864_2015_2138_MOESM1_ESM.pdf]

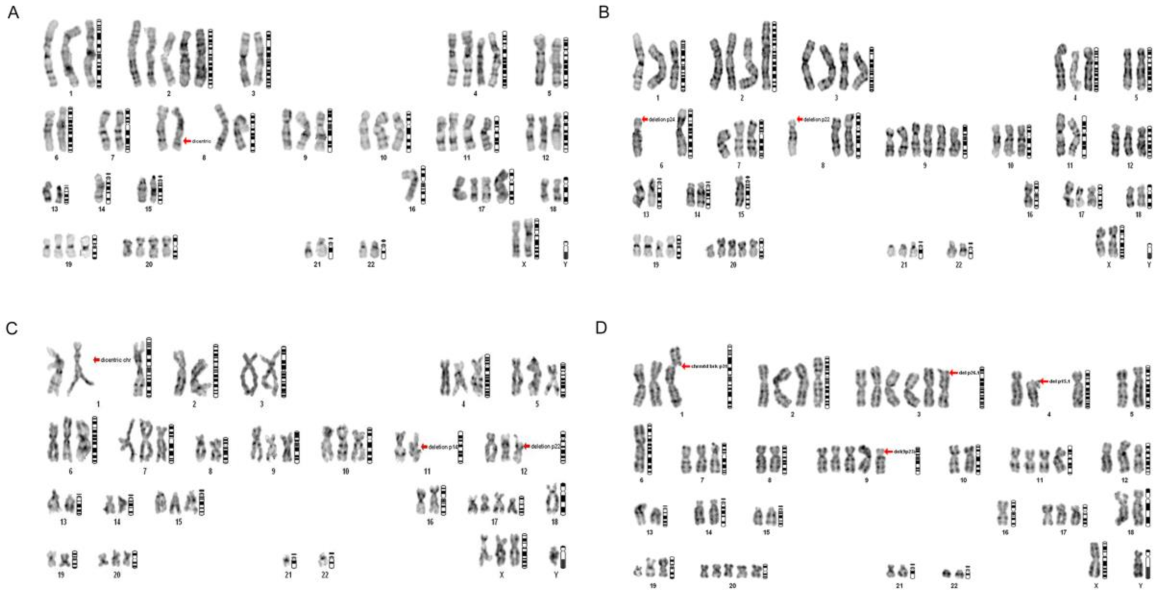

Figure S1. Chromosomal aberration in HNSCC patient derived cell lines AW8507, AW13516, NT8e and OT9. (A) Representative karyotype of AW8507, AW13516, NT8e and OT9 cells is shown from total 25 karyotypes obtained per cell line. (B) Chromosomal aberrations identified by 25 independent karyotype of each cell line is represented in circular form. Chromosome numbers in each cell line are indicated by n, as observed from karyotype (*) and predicted by SNP array (^).

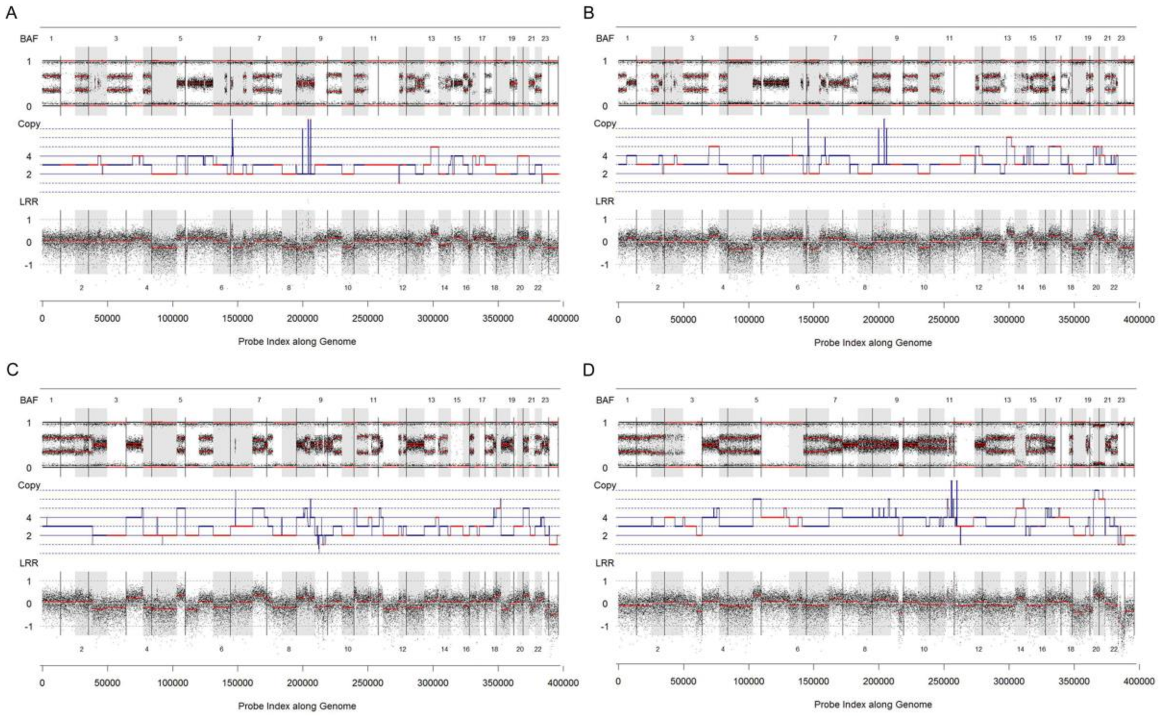

Figure S2. Copy number changes in HNSCC cell lines identified by SNP array. Genome alteration print (GAP) of (A) AW8507, (B) AW13516, (C) NT8e AND (D) OT9 cell lines obtained by SNP array. First horizontal block represents B-allele frequency, second block represents absolute copy number, third block is log R ratio.

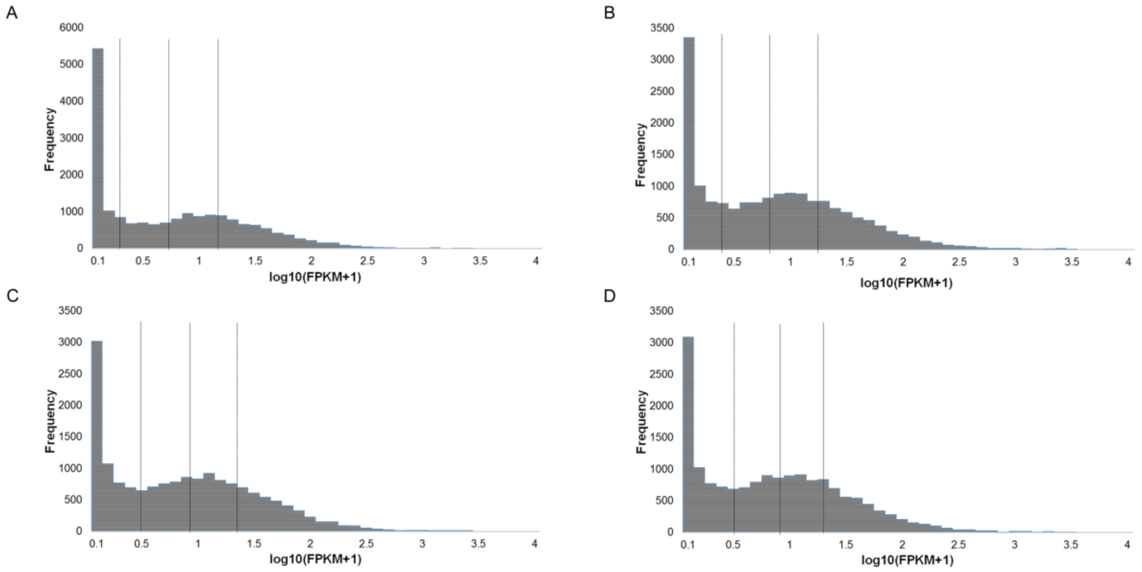

Figure S3. Frequency of transcripts per binned log transformed FPKM + 1. Raw RNA sequencing data was binned to obtain frequency of genes per log10(FPKM + 1) in (A) AW8507, (B) AW13516, (C) NT8e and (D) OT9. Horizontal dotted lines indicates percentile of transcripts in the quadrant.

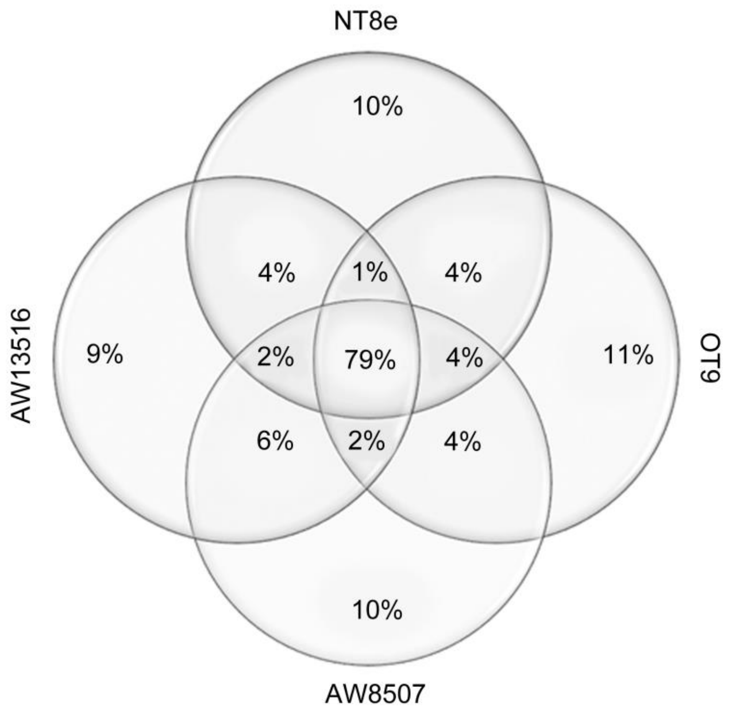

Figure S4. Similarity of gene expression in HNSCC cell lines. Number of genes commonly expressed between AW8507, AW13516, NT8e and OT9 cell lines.

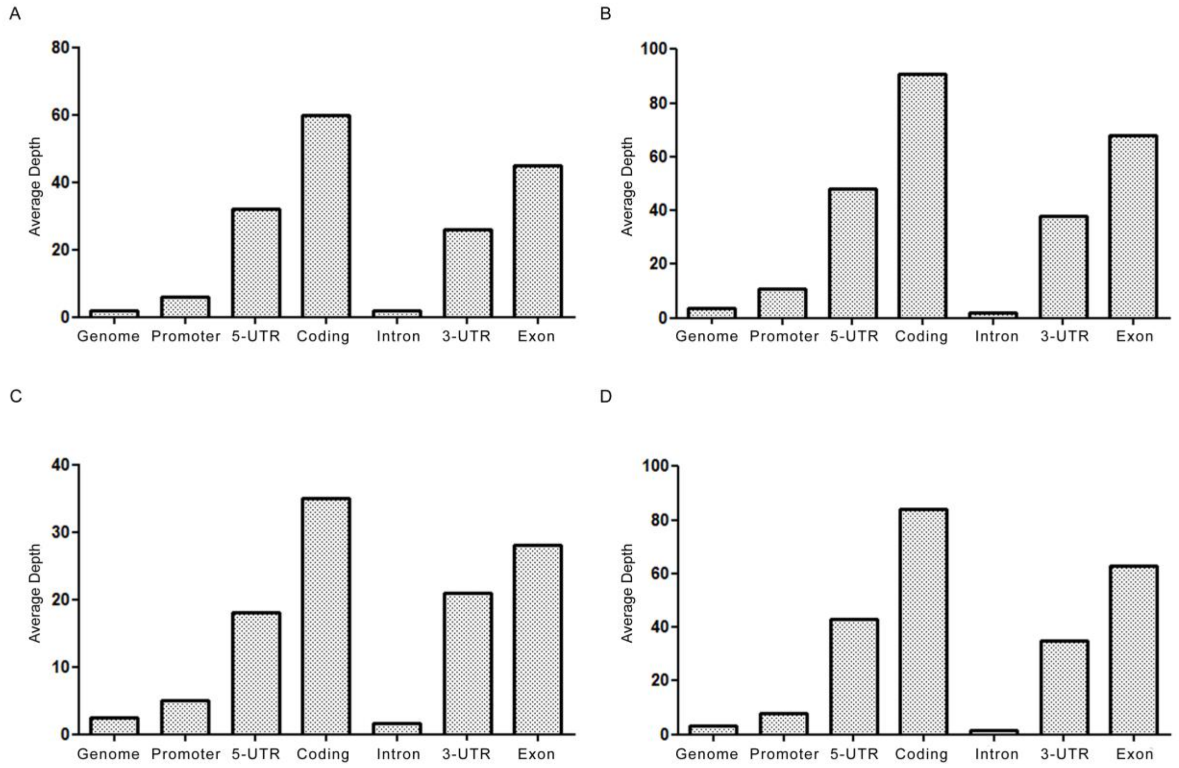

Figure S5. Relative depth in exome sequencing. Relative depth of sequencing for various genomic regions in (A) AW8507, (B) AW13516, (C) NT8e and (D) OT9.

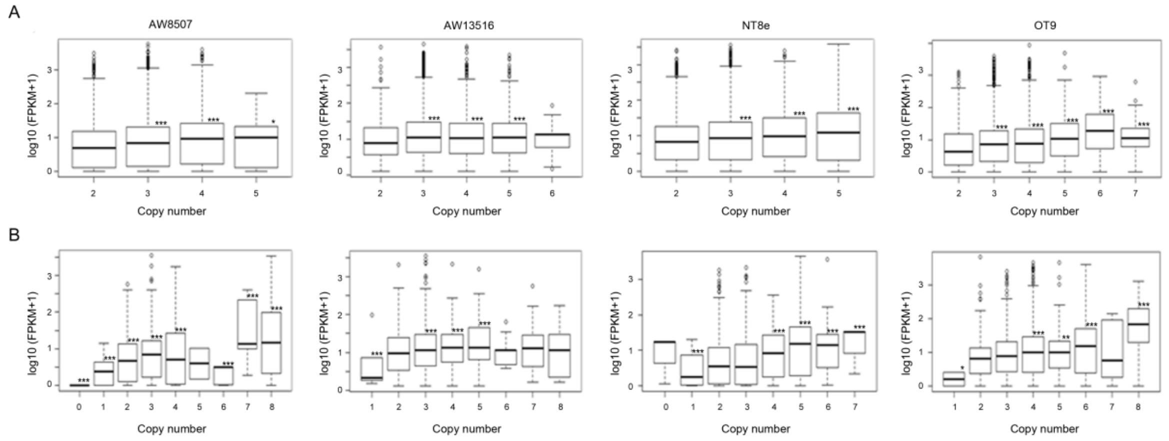

Figure S6. Correlation of copy number with gene expression. (A) Arm level and (B) focal copy number changes and gene expression (y-axis) are shown for AW8507, AW13516, NT8e and OT9 cell lines. Correlation of focal copy number with gene expression was 1.5 fold higher in AW8507, 5.2 fold higher in AW13516, 2.4 fold higher in NT8e and 1.6 fold higher in OT9 cell line compare to arm level copy number changes. P-value cut-off of 0.05 was used as threshold for statistical significance.* denotes P-value <0.05, ** <0.005, *** <0.0005.

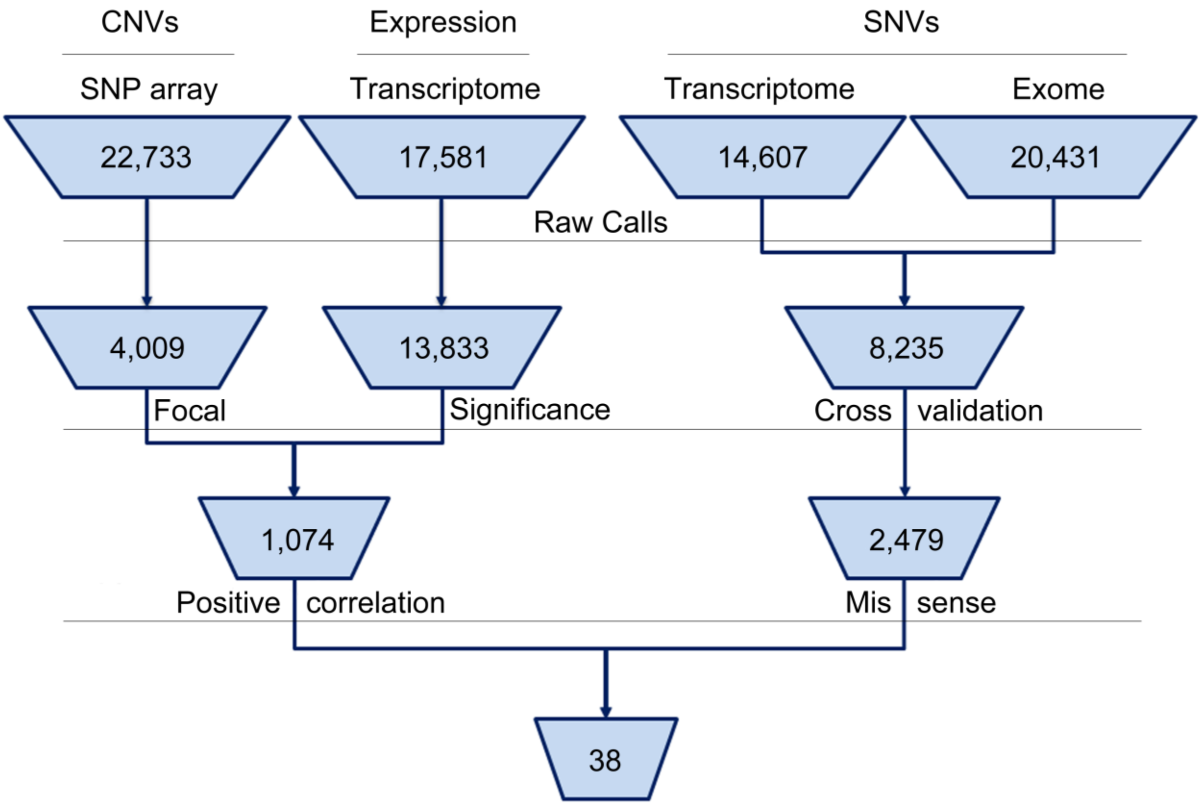

Figure S7. Schematic view of data reduction in integrated genomic analysis. Flow chart depicts the reduction of data at each stage of integration. First row indicates number of genes identified from each platform as raw calls. Second and third row indicates number of genes left after each step of integration with main selection parameter indicated outside the box.

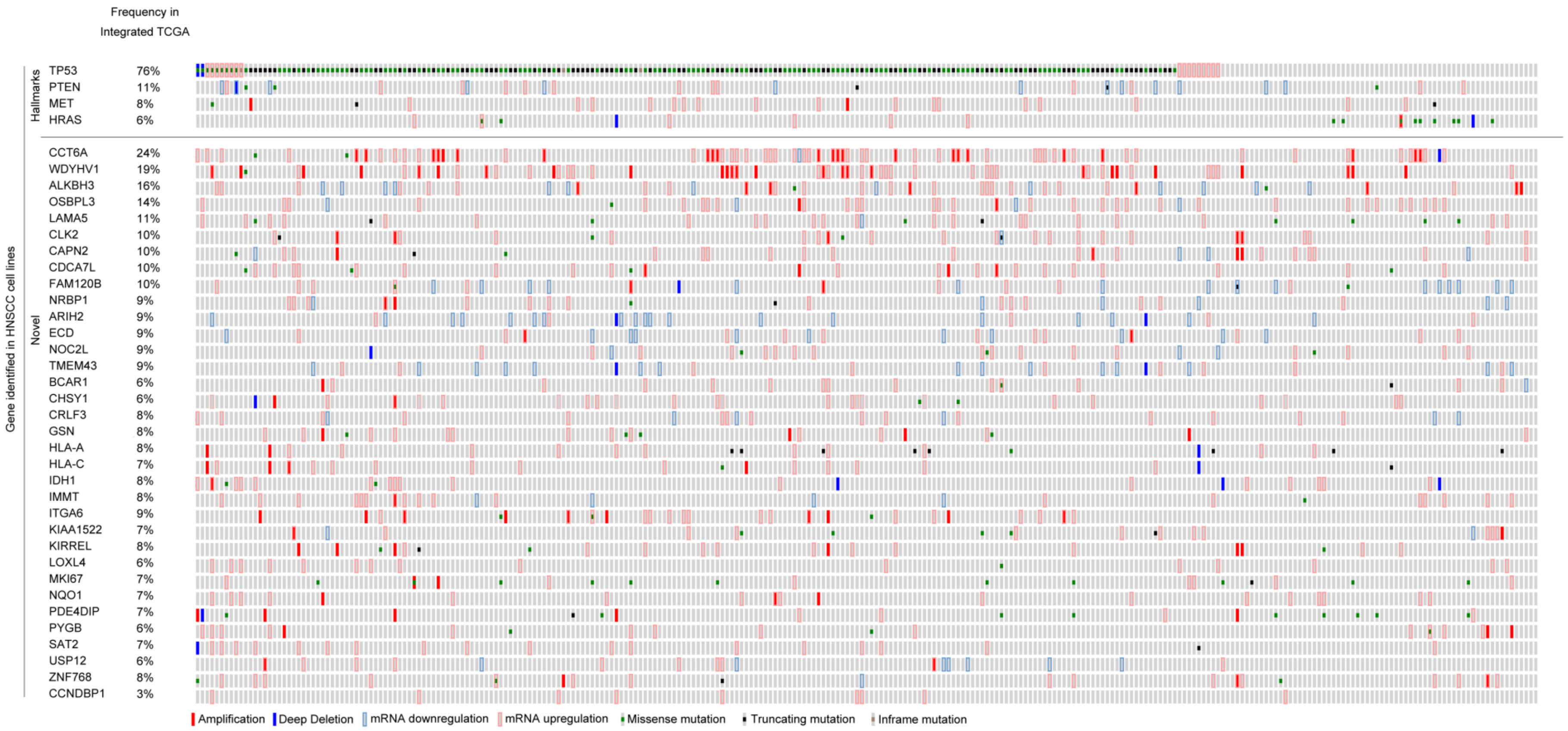

Figure S8. Integrative genomic alterations of genes in TCGA dataset of HNSCC tumors. Heatmap representation of 38 genes in 279 HNSCC samples from TCGA study with frequency of alterations based on integrated CNVs, gene expression and SNVs. Amplification (red) and deletions (blue) are indicated by filled box, over expression (red) and under expression (blue) are indicated by border line to the box, mis-sense (green), non-sense (black) and in-frame (brown) mutations are indicated by smaller square box.

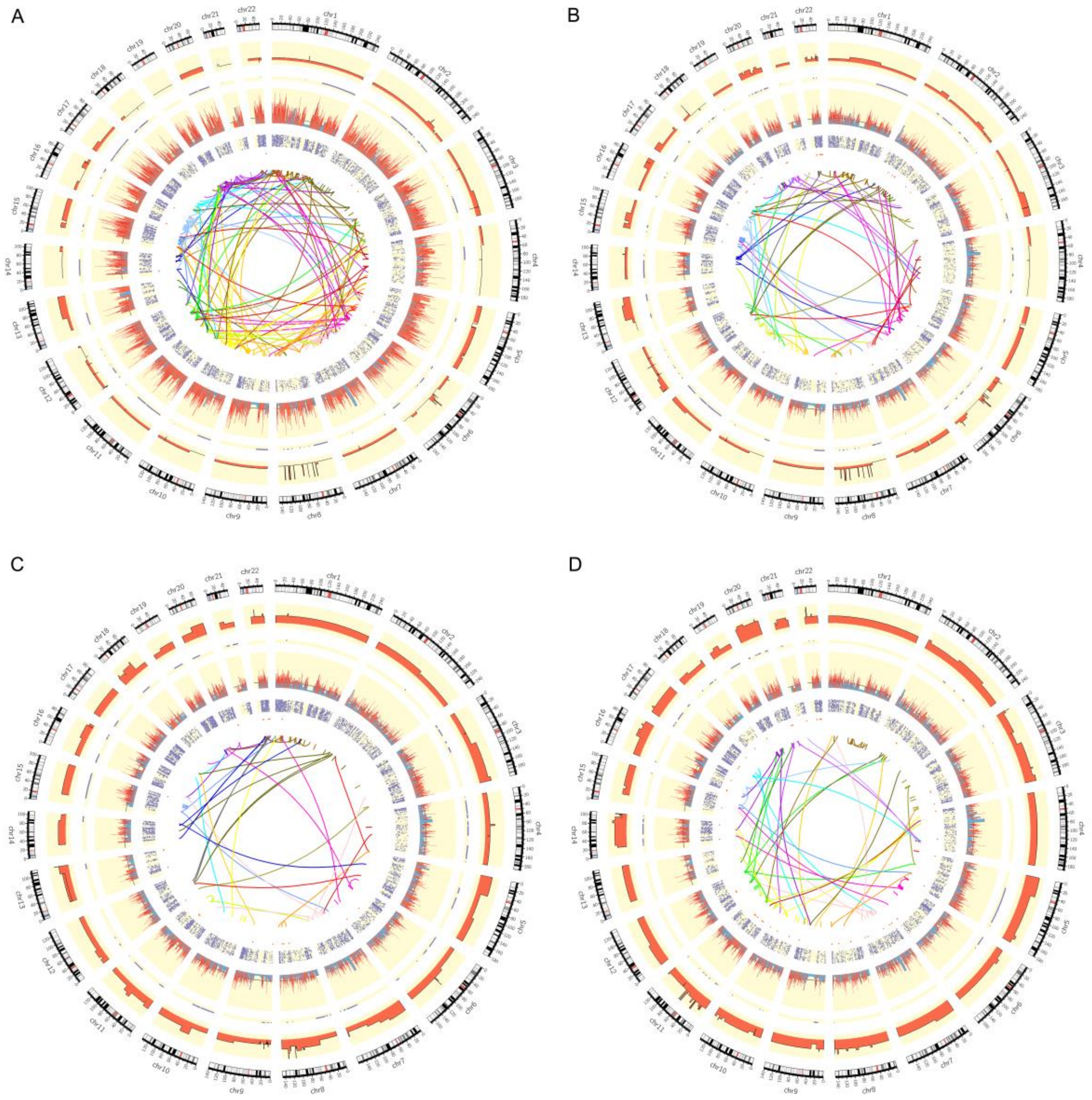

Figure S9. Circos plot representation of HNSCC cell lines. Circos plot representations of integrated genomic data of (A) AW8507, (B) AW13516, (C) NT8e and (D) OT9 cell lines. From outside to inside: karyotype, CNVs, Gene expression (FPKM), SNVs and translocations. Red colour indicates copy number gain or higher gene expression and blue colour indicates copy number loss or lower gene expression in CNV and FPKM tracks, respectively. Non-synonymous mutations are indicated as blue triangles and grey circles represents non-sense mutations in SNV track. Fusion transcripts identified by transcriptome sequencing are shown as arc coloured by their chromosome of origin identified by ChimeraScan.

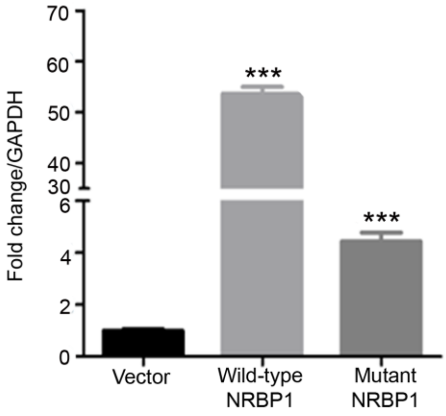

Figure S10. NRBP1 expression in NIH-3 T3 cells. qPCR analysis of NRBP1 gene expression in NIH-3 T3 stably expressing wild and mutant. Data was normalized against GAPDH and fold change plotted. P value <0.0001 is denoted as ***.
