# Supplementary material for: Integrated genomics approach to identify biologically relevant alterations in fewer samples
Source: BMC Genomics. 2015 Nov 14;16:936. doi: 10.1186/s12864-015-2138-4 (PMC4647579; doi:10.1186/s12864-015-2138-4)
Supplement: Additional file 2: — Additional Tables S1-S8: Copy number alterations of known genomic locations identified in HNSCC cell lines. Copy number alterations in halmark genes identified in HNSCC cell lines. Gene expression of hallmark genes by RNA sequencing and qPCR. Features of whole exome and transcriptome sequencing. Validation of mutations in hallmark and novel genes. Details of mutations identified by integrated analysis in HNSCC cell lines. Primer sequences used for Sanger sequenicng based validation of mutations. Primers used for copy number and gene expression study using qPCR. (ZIP 249 kb) [file 12864_2015_2138_MOESM2_ESM.zip › Pratik et al_SI_Tables_20150910.pdf]

Table-S1: Copy number alterations of known genomic locations identified in HNSCC cell lines.

**Alteration**		**Observed in our analysis**
Copy Number Gains	2q	AW8507, AW13516, OT9
	3q	AW8507, AW13516, NT8e
	5p	AW8507, NT8e, OT9
	7p	AW13516, NT8e, OT9
Copy Number Loss	3p	NT8e, OT9
	9p	NT8e, OT9
	10p	AW8507, AW13516, NT8e
	11q	OT9
	14q	NT8e
	17q	AW13516
	19p	AW8507, AW13516, NT8e

Table-S2: Copy number alterations in halmark genes identified in HNSCC cell lines.

	SNP array copy number				qPCR validation (Typeit)			
	NT8e	AW13516	AW8507	OT9	NT8e	AW13516	AW8507	OT9
CCND1	4	3	3	8	3	34	3	5
MET	2	3	3	4	2	21	2	3
MYC	6	7	8	4	2	8	4	2
PIK3CA	4	5	4	4	2	6	3	2
HRAS	4	3	3	4	4	35	6	6
HES1	5	5	4	5	3	15	7	3
JAK1	3	4	3	3	3	2	6	2
CDKN2A	1	3	3	2	4	1	3	4
DLL3	3	3	2	2	2	27	2	3
FBXW7	2	2	2	3	1	1	3	1
NOTCH1	3	3	3	4	6	3	15	6
NSD1	3	4	4	4	1	1	4	1

Table-S3: Gene expression of hallmark genes by RNA sequencing and qPCR.

	RNA sequencing (log10[FPKM+1])				Real-time qPCR (Ct values)			
Gene	NT8e	OT9	AW13516	AW8507	NT8e	OT9	AW13516	AW8507
GAPDH	3.3	3.3	3.5	3.3	17.2	16.5	16.6	20.7
HRAS	2.2	1.9	2.0	2.0	22.7	23.7	23.5	27.6
MET	1.6	2.1	1.6	1.7	22.5	21.3	21.1	26.4
JAK1	1.5	1.5	1.4	1.4	22.1	21.6	21.6	29.5
CDKN2A	1.2	2.0	2.1	1.5	25.4	25.1	23.9	29.0
NSD1	0.9	0.8	0.8	1.1	22.2	22.7	22.4	31.8
FBXW7	0.8	0.7	0.6	0.7	25.1	26.0	26.0	30.2
SMAD4	0.7	0.7	0.8	0.8	23.9	26.0	23.9	27.5

Table-S4: Features of whole exome and transcriptome sequencing.

Cell Line	AW8507	AW13516	NT8e	OT9
Number of mapped reads (% of total reads)	66,251,943 (97.66%)	78,224,226 (93.69%)	49,728,777 (93.95%)	68,206,969 (94.54%)
Average coverage of coding region	50	88	84	81
Total variants in exome sequencing (Ti/Tv)	47892 (2.21)	28813 (2.19)	20864 (2.04)	25029 (2.17)
Total variants in transcriptome sequencing (Ti/Tv)	53330 (1.03)	44168 (1.19)	109609 (1.07)	63458 (1.03)
Non-synonymous variants	5623	4498	2775	5139
Mean number of non-synonymous variants per Mb coding DNA	90	72	44	82

Table-S5: Validation of mutations in hallmark and novel genes.

Hugo Symbol	Nucleotide Change	Protein Change	Validation Status	Cell lines	Cosmic Tissue Type
ASPM	C>G	C195S	Validated	NT8e	Novel
CAPN2	C>A	R461S	Validated	OT9	Skin
CASP8	G>A	G328E	Validated	AW13516	Melanoma
DCC	G>A	R667H	Validated	NT8e	HNSCC
EGFR	G>A	R521K	Validated	OT9	HNSCC
FLG	C>T	D2339N	Validated	NT8e	Novel
HRAS	C>T	R68W	Validated	AW13516	HNSCC
HRAS	G>A	G12S	Validated	AW13516,AW8507	Novel
MET	A>G	N375S	Validated	AW13516	Lung
NETO1	G>A	G402R	Validated	NT8e	Novel
NRBP1	C>T	Q73*	Validated	NT8e	Lung
PAK6	C>T	R675*	Validated	NT8e	HNSCC
PTEN	C>T	H141Y	Validated	OT9	Glioma
SLFN5	C>T	R632W	Validated	OT9	Endometrium
TP53	C>G	P72R	Validated	AW13516	HNSCC
TP53	G>A	R273H	Validated	AW13516	Bladder, Stomach
ZNF594	G>T	K758N	Validated	NT8e	Novel
NLRP2	C>T	T598M	Invalidated	NT8e	Novel
TACC2	C>T	S408F	Invalidated	AW8507	Novel
TRIM42	C>G	C68W	Invalidated	NT8e	Novel

Table-S7: Primer sequences used for Sanger sequenicng based validation of mutations.

S.NO.	Primer ID	Sequence(5'-------------->3')
1	OAD1119_*EGFR*_F	CCTCCACTGTCAGGCACATTTC
2	OAD1120_*EGFR*_R	GGATTATATTAGGCAATAATAC
3	OAD1121_*MET*_F	ACACAAGAATAATCAGGTTCTG
4	OAD1122_*MET*_R	CTTTTAGTTACCATTTACATTTTA
5	OAD1123_*PTEN*_F	CTCTGGAATCCAGTGTTTCTTT
6	OAD1124_*PTEN*_R	ATAATTATGTGAGGTGATGAAT
7	OAD1125_*TP53*_R273H_F	TTCCACTTGATAAGAGGTCCC
8	OAD1126_*TP53*_R273E_R	CTTACCGATTTCTTCCATACT
9	OAD1127_*TP53*_P72R_F	CACTGACAGGAAGCCAAAGGG
10	OAD1128_*TP53*_P72R_R	CTGTGGGAAGCGAAAATTCCA
11	OAD1129_*CASP8*_F	GGTCAAATTCTTATCTATCAAT
12	OAD1130_*CASP8*_R	CTCTGGCAAAGTGACTGGATG
13	OAD24_*DCC*_F	GCAAAGGAGAGGTGATAATGCATTC
14	OAD25_*DCC*_R	CCAATAGATGTCACCTGCCTGG
15	OAD26_*PAK6*_F	CCTGCTCCATAGGGGACTTGGC
16	OAD27_*PAK6*_R	CCAGGATGTTCTGCCATTGTGG
17	OAD30_*UBE2O*_F	CAGCTGGGAGACGGACAATG
18	OAD31_*UBE2O*_R	GTTGGCTTCTCAGGCTCCAC
19	OAD36_*FLG*_F	GCTCAGGAGCAGTCAAGAGATG
20	OAD37_*FLG*_R	GTCCAGACCTTCCTGCTGAC
21	OAD55_*UNC5C*_F	CTGGGTTGTTGATCTATGGCATC
22	OAD56_*UNC5C*_R	GGATTACAGGCGTGAGTCACCG
23	OAD676_*HRAS*_G12S_F	ATGACGGAATATAAGCTGGTG
24	OAD678_*HRAS*_G12S_R	CTGTACTGGTGGATGTCCTC
25	OAD689_*HRAS*_R68W_F	CATCCAGGACATGCGCAGA
26	OAD690_*HRAS*_R68W_R	CCCTGTCTCCTGCTTCCTCT
27	OAD79_*ZNF594*_F	GAATGTGGGAATGCCTTCAGGCG
28	OAD80_*ZNF594*_R	GTGTGTGACAAGGTGGGACCTC
29	OAD85_*NETO1*_F	CCTGCTGGACCAGCTGACCAAC
30	OAD86_*NETO1*_R	GGCTGTGTGGGCATCTCTGTC
31	OAD96_*CAPN2*_F	GGGCTTCCTGTGTTGCCCAG
32	OAD97_*CAPN2*_R	GGCCGAGGAACAGACCAGTG
33	OAD98_*NRBP1*_Q73*_F	GCTGGCAGAAGAGGCGAGAAG
34	OAD99_*NRBP1*_Q73*_R	GAAGCCATTCCCTATCCCTCC

Table-S8: Primers used for copy number and gene expression study using qPCR.

Application	S.NO.	Primer ID	Sequence(5'-------------->3')
COPY NUMBER AND EXPRESSION	1	OAD1061_*MET*_F	TTCTGACCGAGGGAATCATCA
	2	OAD1062_*MET*_R	CCTTCACTTCGCAGGCAGAT
	3	OAD1051_*JAK1*_F	CTTTGCCCTGTATGACGAGAAC
	4	OAD1052_*JAK1*_R	ACCTCATCCGGTAGTGGAGC
	5	OAD1059_*CDKN2A*_F	CAAGATCACGCAAAAACCTCTG
	6	OAD1060_*CDKN2A*_R	CGACCCTATACACGTTGAACTG
	7	OAD1067_*FBXW7*_F	CCACTGGGCTTGTACCATGTT
	8	OAD1068_*FBXW7*_R	CAGATGTAATTCGGCGTCGTT
	9	OAD1049_*NSD1*_F	TCCTGAGTCAGAACATGACCTG
	10	OAD1050_*NSD1*_R	CGAGATTTAGCGCAAGGCTTTT
EXPRESSION	11	OAD67_*GAPDH*_F	AATCCCATCACCATCTTCCA
	12	OAD68_*GAPDH*_R	TGGACTCCACGACGTACTCA
	13	OAD1065_*SMAD4*_F	GCTGCTGGAATTGGTGTTGATG
	14	OAD1066_*SMAD4*_R	AGGTGTTTCTTTGATGCTCTGTCT
	15	OAD1131_*HRAS*_F	TTTGAGGACATCCACCAGTACA
	16	OAD1132_*HRAS*_R	GCCGAGATTCCACAGTGC
COPY NUMBER	17	OAD1057_*NOTCH1*_F	GTGACTGCTCCCTCAACTTCAAT
	18	OAD1058_*NOTCH1*_R	CTGTCACAGTGGCCGTCACT
	19	OAD1063_*HRAS*_F	CGGCAGGGAGTGGAGGAT
	20	OAD1064_*HRAS*_R	TTCAGCTTCCGCAGCTTGT
	21	OAD1069_*CCND1*_F	GAACTACCTGGACCGCTTCC
	22	OAD1070_*CCND1*_R	TAGAGGCCACGAACATGCAA
	23	OAD1071_*MYC*_F	AGAGTTTCATCTGCGACCCG
	24	OAD1072_*MYC*_R	AAGCCGCTCCACATACAGTC
	25	OAD 939_*PIK3CA*_F	TATTTGCTTTTTCTGTAAATCATC
	26	OAD 940_*PIK3CA*_R	GCTTTGAGCTGTTCTTTGTCAT
	27	OAD502_*HES1*_F	AGGGCGTTAATACCGAGGTG
	28	OAD503_*HES1*_R	AGGTCATGGCATTGATCTGGG
	29	OAD282_*DLL3*_F	CCCTACCCTTCCTCGATTCTG
	30	OAD283_*DLL3*_R	GAACTGAAAATGGGCTTAAAACCTT
